# FIZ1 is part of the regulatory protein complex on active photoreceptor-specific gene promoters *in vivo*

**DOI:** 10.1186/1471-2199-9-87

**Published:** 2008-10-14

**Authors:** Raghuveer S Mali, Guang-Hua Peng, Xiao Zhang, Loan Dang, Shiming Chen, Kenneth P Mitton

**Affiliations:** 1 Eye Research Institute, Oakland University, Rochester, MI, USA; 2 Department of Ophthalmology and Visual Sciences, Washington University School of Medicine, St. Louis, MO, USA; 3 Department of Developmental Biology, Washington University School of Medicine, St. Louis, MO, USA

## Abstract

**Background:**

FIZ1 (Flt-3 Interacting Zinc-finger) is a broadly expressed protein of unknown function. We reported previously that in the mammalian retina, FIZ1 interacts with NRL (Neural-Retina Leucine-zipper), an essential transcriptional activator of rod photoreceptor-specific genes. The concentration of FIZ1 in the retina increases during photoreceptor terminal maturation, when two key transcription factors NRL and CRX (Cone-Rod Homeobox) become detectable on the promoters of photoreceptor-specific genes (i.e. *Rhodopsin, Pde6b*). To determine if FIZ1 is involved in regulating CRX-mediated transcriptional activation, we examined FIZ1 subcellular location in mouse neural retina, its ability to interact with CRX, and its association with CRX/NRL target genes.

**Results:**

FIZ1 is present in the nucleus of adult photoreceptors as well as other retinal neurons as shown by transmission electron microscopy with nano-gold labeling. FIZ1 and CRX were co-precipitated from retinal nuclear extracts with antibodies to either protein. Chromatin immunoprecipitation (ChIP) assays revealed that FIZ1 is part of the protein complex on several rod and cone gene promoters, within photoreceptor cells of the mouse retina. FIZ1 complexes with CRX or NRL on known NRL- and CRX-responsive elements, as shown by electrophoretic mobility shift assays with FIZ1 antibody. FIZ1 can directly bind to CRX, as demonstrated using yeast two-hybrid and GST pull-down assays. Co-transfection assays demonstrated that FIZ1 increases CRX-mediated activation of *Opsin *test promoters. Quantitative ChIP analysis revealed an increased association of FIZ1 with the *Rhodopsin *promoter in adult (P-25) neural retina versus immature (P-3) neural retina. The quantity of transcriptionally active RNA Polymerase-II within the *Rhodopsin *gene (*Rho*) was significantly increased in the adult neural retina, compared to the immature retina.

**Conclusion:**

FIZ1 directly interacts with CRX to enhance CRX's transactivation activity for target genes. Developmentally, in neural retina tissue, the increased association of FIZ1 with CRX target genes corresponds to an increased association of transcriptionally active Pol-II within the *Rho *gene. Together with previous findings, our results suggest that FIZ1 may act as a transcriptional co-regulator of photoreceptor-specific genes, recruited by at least two photoreceptor-specific transcription factors, CRX and NRL. Further studies are underway to elucidate the exact role of FIZ1 in photoreceptor gene expression, development and maintenance.

## Background

The mammalian retina is an excellent paradigm to study both differentiation and maturation of neurons from common precursor cells [1]. Several transcription factors are clearly essential for photoreceptor development, and their mutations cause retinal degenerations: NRL, CRX, Otx2, Trβ2 (thyroid hormone receptor β2), and NR2E3 [2-6]. NRL, CRX and NR2E3 are present in photoreceptor progenitors quite early (E-14-18 in the mouse) when they influence photoreceptor type. While present in photoreceptor progenitors, they are not associated with the *Rhodopsin *(*Rho*) promoter *in vivo*, until these neurons begin to mature and *Rho *is expressed [7]. Regulators of photoreceptor-specific gene promoters appear to form a transcriptional complex supported by their multiple protein-protein interactions. NRL and CRX can interact directly with each other, and they synergize to activate the *Rho *promoter *in vitro *[8]. Likewise, NR2E3 interacts with CRX, and supports CRX-mediated activation of rod-specific gene promoters, and repression of cone-specific gene promoters [9,10].

Through efforts to map protein interactions involved in regulating retinal gene expression, FIZ1 was found as a protein that interacts with NRL, and co-purifies with NRL from nuclear extracts [11]. While FIZ1 mRNA is found in most human and mouse tissues, FIZ1 protein content is lower in immature postnatal mouse retina and its concentration increases ten-fold (after P-5) as retinal neurons mature to full functionality [11,12]. Immunohistochemistry has revealed increasing FIZ1 concentration in photoreceptors, the inner plexiform layer, and the ganglion cell layer [13]. Functionally, FIZ1 can synergize with NRL and CRX to increase the activation of the *Rho *and *PDE6B (human rod Phosphodiesterase beta subunit) *promoters *in vitro *[13].

Is FIZ1 recruited to the regulatory protein complex on the *Rhodopsin *promoter *in vivo*? To address this question, we wanted to determine the subcellular localization of FIZ1 in the neural retina at the ultrastructural level. We utilized transmission electron microscopy (TEM) with nano-gold labeling to accomplish this goal. To determine if FIZ1 is part of the protein complex, with NRL and CRX, on photoreceptor specific gene promoters *in vivo*, we employed electrophoretic mobility shift assays (EMSA) with retinal nuclear extracts, and chromatin immunoprecipitation (ChIP) assays.

Knowing that members of these transcriptional complexes (i.e. NRL, CRX, NR2E3) are typically involved in several protein-protein interactions, we carried out a set of assays to determine if FIZ1 could interact with CRX. These included yeast two-hybrid assays, GST pull-down assays, co-immunoprecipitation assays, and promoter activation assays with two test *Opsin *promoters (*M- *and *S-Opsin*). Lastly, we employed quantitative ChIP (Q-ChIP) assays to compare the association of FIZ1 with the *Rho *gene in immature retina (poised to transcription) versus mature retina (actively transcribed). A novel technique to directly monitor the transcriptional state of the *Rho *gene, based upon the presence of transcriptionally active RNA Polymerase-II within the gene, was adapted successfully for tissue analysis.

We report that FIZ1 is present in the nucleus of rod and cone photoreceptors as well as other mature retinal neurons. FIZ1 is part of the regulatory protein complex associated with active rod- and cone-specific gene promoters, but only in photoreceptor cells. Furthermore, FIZ1 can bind directly to CRX, and modify CRX's activation potential at two *Opsin *(M and S) promoters *in vitro*. Q-ChIP revealed that FIZ1 association with the *Rho *gene increases in maturing photoreceptors and this correlated with a greater amount of actively transcribing RNA Polymerase-II moving through the *Rho *gene.

## Methods

Studies were carried out with the approval of Oakland University's Animal Care and Use Committee and Washington University's Animal Studies Committee. This research complied with the Statement for the Use of Animals in Ophthalmic and Vision Research, as adopted by the Association for Research in Vision and Ophthalmology.

### Nano-gold Labeling and Transmission Electron Microscopy

Nano-scale immunogold labeling was performed according to previously described methods that yield good sensitivity with mouse neural retina [14]. Fixation, resin type, and sectioning, were optimized to preserve sufficient retinal morphology, with minimal masking of antigen. Mouse retinas, immature (P-5) and mature (P-35), were fixed with 4% paraformaldehyde in 0.1 M phosphate buffer (pH 7.4) for 4 hours and washed with phosphate buffered saline (PBS, pH 7.4) over night. Retinas were dehydrated with ascending N, N-dimethyl formamide and infiltrated with Lowicryl-K4M (Ted Pella Inc., Redding, CA) [15]. Samples were oriented in gelatin capsules and polymerized under UV light for 3 hours. Thin sections (70 nm) were cut using an RMC ultra microtome MT 7000 and picked up on formvard-coated grids. Grids with retinal sections were floated with 1 drop of saturated sodium meta periodate for 1 hour at room temperature and washed with PBS containing 0.2% Tween-20 (PBS-T, 3 × 5 min). Samples were incubated with 0.1 M glycine solution for 15 minutes and washed with PBS-T (3 × 5 min). Samples were blocked for non-specific protein binding with 5% normal goat serum in PBS-T for 1 hour and incubated with anti-bFIZ1 antibody (1:100, 4°C), a rabbit IgG made to target domain-II-IV of FIZ1 (Proteintech Group Inc., Chicago, IL, USA) that was affinity purified and specific for FIZ1 in immunoblots of mammalian retina [11,13]. After washing with PBS-T (3 × 5 min), samples were incubated with Nanogold-Fab'goat anti-rabbit-IgG nano gold (gold particle size 1.5 nm maximum, 1.4 nm average), 1:50 in PBS-T, 1 hour at 37°C (Nanoprobes, Yaphank, NY). Samples were washed with PBS-T (3 × 5 min) and post-fixed with 1% glutaraldehyde in PBS for 10 minutes. After washing with PBS-T (3 × 5 min) followed by distilled water, nano-gold particles were enhanced with silver, and samples were double stained with uranyl acetate and lead citrate [15]. Digital photographs of samples were captured using a Philips Morgani™-268 Transmission Electron Microscope, fitted with FEI Electron Optics (Eindhoven, The Netherlands).

### Co-immunoprecipitation (Co-IP) from bovine retina nuclear extracts

Preparation of bovine retina nuclear extract and Co-IP were accomplished as previously described [11,16]. Briefly, bovine retinal nuclear extract (300 μg protein) was incubated with anti-bFIZ1 or anti-CRX polyclonal antibodies overnight at 4°C with gentle mixing. Both antibodies were specific for FIZ1 or CRX in immunoblots of neural retina. Anti-bFIZ1 was the rabbit IgG, targeted to domain-II-IV of FIZ1 (Proteintech Group Inc., Chicago, IL, USA). Anti-CRX was an affinity purified rabbit polyclonal antibody (Anti-CRX-p119b) targeting amino acids 119–141 of mouse CRX (Proteintech Group Inc., Chicago, IL, USA) [7]. The immunoprecipitates were recovered with protein A-Sepharose (GE Healthcare), washed with PBS, 1% Triton X-100, and proteins subjected to SDS-PAGE and immunoblotting with the appropriate antibodies: anti-bFIZ1 (1:3000) or anti-CRX (1:2000). Blots were visualized using the ECL plus detection reagents (GE Healthcare).

### Chromatin Immuno-Precipitation (ChIP)

Basic ChIP PCR assays were performed as described previously [17]. The mouse strains used were: C57BL/6J (6–8 weeks old) referred to as wild-type, and C57BL/6J-*Pdeb*^*rd1 *^*le/Pdeb*^*rd1 *^*le *(P-90, The Jackson Lab) referred to as "rodless/coneless" [18,19]. FIZ1 associated chromatin was immunoprecipitated from wild-type retina using anti-bFIZ1 antibody which is specific for FIZ1 protein in immunoblots of mouse retina. ChIP DNA was analyzed by PCR with primers targeting the proximal promoter regions of the following genes: *Rho, Pde6b, M-Opsin, S-Opsin, Rbp3*, and *Alb *[17]. Primer sequences are listed in the Table 1. Input chromatin DNA served as a positive control, while pre-immune serum, no antibody and no DNA (mock) samples served as negative controls. Follow up ChIP assays compared FIZ1 association with gene promoters in retinas from wild-type and rodless/coneless mice. The antibody was anti-FIZ1-I, an affinity-purified rabbit IgG that targets a conserved peptide (mouse and human) in domain-I (Proteintech Inc., Chicago, IL, USA), and detects the same band as anti-bFIZ1 on immunoblots (data not shown). ChIP DNA was analyzed by PCR, targeting the several photoreceptor-specific promoters: *Rho, Pde6b, M-Opsin, S-Opsin, and Rbp3*. Analysis included the mGluR6 promoter, as a non-photoreceptor neuron control.

**Table 1:** PCR primers for chromatin immunoprecipitation assays

**Gene**	**Positions†**	**Sense primer**	**Anti sense primer**	**bp**
*Rho*	-200 to +64	ggggcagacaagatgagacac	ttcgtagacagagaccaaggc	264

*Pde6b*	-201 to +56	actgcccataactcctgtaac	tgttcctgctgctgtgcccg	257

*Rbp3*	-28 to +95)	cctcacatctaactcccacattg	ccttggctcctggataagag	123

*Mop*	-216 to -23	tgagccacccctgtggattg	ggaacctgtcagacttggcac	194

*Sop*	-396 to -202	cactcatcctcttcctgtttcc	ggtcagtattggtttctgtggc	195

*Alb*	-547 to -82	ggacacaagacttctgaaagtcctc	ttcctaccccattacaaaatcata	465

*mGluR6*	-139 to +67	ccagaggttggctcaggtaag	gcaggaaaagttggtgactcg	206

†Relative to the transcriptional start site (+1).

*Rho *(Rhodopsin), *Pde6b *(cGMP-dependent phosphodiesterase-beta subunit), *Rbp3 *(Interphotoreceptor retinoid binding protein), *Mop *(M-Opsin), *Sop *(S-Opsin), *Alb *(Albumin), *GluR6 *(metabotropic glutamate receptor subtype-6).

### Electrophoretic mobility shift assays (EMSA)

EMSA was performed with a gel-shift assay system according to the manufacturer's instructions (Promega). Nuclear extracts from bovine retina were prepared as previously described [11,16,20]. Short double-stranded oligonucleotides, for probes, were synthesized for three conserved cis-elements from the mammalian *Rhodopsin *proximal promoter region that are known to bind NRL or CRX: the NRL-response element, NRE, (5'-CAGATGCTGATTCAGCCAGGAGCT)[21]; the CRX-binding element, BAT-1, (5'-GCAGCAGTGAGGATTAATATGATTAATAACG)[5]; and the CRX-binding element, Ret-4, (5'-GGGAGCTTAGGGAGGGGAGG)[5]. These probes were end-labeled with T4 polynucleotide kinase (10 Units/μL) and [γ-^32^P]ATP (3000 Ci/mmol at 10 mCi/mL). ^32^P-labeled probes (20 fmoles, 10^5 ^cpm) were incubated with retinal nuclear extract (5 μg protein) in binding buffer (20 mM Tris-HCl, pH 7.5, 100 mM NaCl, 2 mM MgCl2, 1 mM EDTA, 1 mM DTT, 1 μg poly-dI-dC, 8% glycerol) for 30 minutes at 21°C. For competition controls, unlabeled ("cold") probe (100x) was incubated with nuclear extracts for 30 minutes at room temperature before addition of ^32^P-labeled oligonucleotide. For FIZ1 antigen-antibody tests, antibody to FIZ1 (anti-GST-bFIZ1) or control (preimmune rabbit IgG serum) was added after the incubation of ^32^P-labeled oligonucleotide with nuclear extracts. The samples were loaded on a pre-run (150 volts, 30 min) 4% non-denaturing polyacrylamide gel (1 mm, 16 cm × 18 cm) in 0.5% TBE, at 150 volts for 5 hours. After electrophoresis, the gel was dried and exposed to X-ray film (Pierce) for autoradiography.

### Yeast two-hybrid assay

The bait plasmid, pHybLex/Zeo-bFIZ1 was prepared by sub-cloning domains II-IV of bovine FIZ1 into the pHybLex/Zeo vector (Invitrogen). The pACT2-bCRX vector provided expression of Gal4-AD-CRX protein. Transformation of L40 yeast and interaction assays were previously described [8]. Bait strains were prepared with the vectors: pHybLex/Zeo-bFIZ1 (expressing LexA-bFIZ1), pHybLex/Zeo-Laminin (LexA-Laminin), and pHybLex/Zeo (LexA). Bait strains were transformed with the desired prey-vector: pACT2-bCRX (Gal4-AD-bovine-CRX) or pACT2 (Gal4-AD).

### GST pull-down assays

The bacterial expression plasmid, pDest15-bFiz II-IV, for preparation of GST-bFIZ1 on glutathione-Sepharose was described previously [11]. For a GST control, pDest15-GST was utilized; a construct prepared by removal of FIZ1 sequence from pDest15-bFiz II-IV. ^35^S-CRX was prepared using the TNT Quick Coupled Transcription/Translation kit (Promega) with ^35^S-methione (> 1000 μCi/mmol; GE Health) and pcDNA3.1/HisC-bCRX as template. GST pull-down assays were carried out as previously described using glutathione-Sepharose-bound GST or GST-bFIZ1 protein (100 μg) in binding buffer (20 mM Tris-Cl, pH 8.0, 150 mM NaCl, 0.2% Nonidet P-40). After washes, the bound proteins were denatured in 60 μL of 2× SDS sample buffer (100°C, 5 min), subjected to SDS-PAGE, and processed for fluorography (GE Healthcare).

### *M*- and *S*-*Opsin *promoter activation assays

Transfection and promoter activation assays were performed as previously described [13]. CV1 cells (obtained directly from the ATCC) were incubated at 37°C, 5% CO_2_, in MEM-alpha with 10% FBS, and penicillin/streptomycin. Expression plasmids for bovine CRX (pcDNA3.1/HisC.bCRX) and bovine FIZ1 (pDest26-bFiz1) were described previously [8,11]. The reporter, *Mop250-Luc*, contained the human *M*-*Opsin *promoter (-253 to +38 bp) sub-cloned into the pGL3-basic vector (Promega) [9]. The reporter *Sop552-Luc*, contained a mouse *S-Opsin *minimal promoter [22] sub-cloned into the pGL3-basic vector (from Dr. Anand Swaroop). Transfection mixes contained 555 ng/mL (on cells) of a reporter plasmid (*Mop250*-Luc, *or Sop552*-Luc) and 19 ng/mL of the *Renilla *Luciferase construct, pRL-CMV (Promega). Triplicate samples (per experiment) were co-transfected with expression vectors for CRX (83 ng/mL) and FIZ1 (165 ng/mL). Firefly- and *Renilla*-Luciferase activities were measured with Dual-Reporter Luminescence Reagent, 48 h post-transfection (Promega). Experiments were repeated three times, and were analyzed independently using ANOVA with Tukey-Kramer post-analysis [13]. Fold activation was relative to the background with empty expression vectors, set as 1-fold.

### Quantitative ChIP (Q-ChIP) assays of FIZ1 and Pol-II Binding

Q-ChIP was performed on normal P-3 (immature) and P-25 (mature) mouse retina to compare FIZ1 association with the *Rho *promoter (Chromosome 6). By P-3, about 85% of rod photoreceptor cells have been "born", and express NRL and CRX, but these immature neurons have not synthesized their phototransduction proteins or their outer-segment structures. An increase of FIZ1 protein association with the *Rho *proximal promoter region should result in a proportional increase in the amount of promoter DNA fragments recovered by ChIP. Real time PCR can be used for quantitative comparison of the DNA recovered (copy numbers), and thus comparison of the amount of FIZ1 binding [23-25]. A non-translated region of Chromosome 6 (*Untr6*) was included as a control. ChIP was performed with antibody to FIZ1 (Anti-bFIZ1). The active state of the *Rho *gene was also measured by Q-ChIP with an antibody to the phospho-serine-2 CTD (C-terminal repeat domain) of RNA-polymerase-II (AbCam Inc., Cambridge, MA). Serine-2 (S2) in the RNA polymerase-II CTD is phosphorylated in the transcriptionally active form of the enzyme [26,27]. Furthermore, the target amplicon for the Pol-II-S2 Q-ChIP assays was situated within intron-II of the *Rho *gene. This location is over 3 Kb downstream of the *Rho *promoter region, to ensure measurement of actively transcribing Pol-II. Primers used for Real-Time PCR are listed in Table 2. Amplicons were also placed to avoid highly repetitive DNA sequences, as determined from mouse genomic data [28] with the UCSC Genome Browser (, University of California, Santa Cruz).

**Table 2:** PCR primers for quantitative chromatin immunoprecipitation assays

**Gene**	**ChIP Antibody**	**Positions†**	**Sense primer**	**Anti sense primer**	**bp**
*Rho*	Pol-II-S2	+3051 to +3199	ggagatcccatgcacaaagt	taagctccctgccacttgac	149

*Rho*	FIZ1	-711 to -570	cagcaggccagtaggatca	gaggaggggcgtaagaagtt	141

*Rho*	FIZ1	-12 to +130	cccctctgcaagccaatta	gcaactccaggcactgac	142

*Untr6*	Pol-II-S2 FIZ1	4.4 × 10^6 ^bp downstream of *Rho*	tcaggcatgaaccaccatac	aacatccacacgtccagtga	216

†Relative to the transcriptional start site (+1).

*Rho *(Rhodopsin), *Untr6 *(untranslated region Chromosome-6), Pol-II-S2

Ct values from triplicate real-time PCR assays were transformed using a standard curve of genomic DNA with known copy numbers, to obtain precipitated copy numbers for each gene test region. To determine the relative PCR efficiency, between different gene regions, triplicate assays were run using non-precipitated genomic DNA (input). Copy numbers of DNA detected were then normalized for the amount of chromatin input, the proportion of ChIP DNA used for Q-PCR, and then for relative primer efficiency. Final results were scaled as copies of DNA detected per 1000 cells (of input DNA).

## Results

### TEM-immunogold labeling of FIZ1 in mature retinal neurons

To visualize the subcellular location of FIZ1 in neural retina, transmission electron microscopy (TEM) with nano-gold labeling was performed on P-5 and P-35 (young adult) mouse neural retina. Photoreceptors in the P-5 retina lack outer-segments and the increased expression of genes required for light detection and visual transduction (i.e. *Rho*) has just started at this time. Retinal neurons are not mature at this time and synaptogenesis has not yet occurred. The retina becomes functional by P-21, when Rhodopsin content has almost reached its adult maximal level. Outer segments (OS) are present at this time, and continue to elongate slightly to about P-31. Fixation and the type of embedding resin were optimized to minimize the antigen masking that occurs in typical plastic embedding for TEM-based morphology analysis. Stage P-5 and P-35 neural retina samples were processed simultaneously throughout the complete procedure.

**Figure 1:**
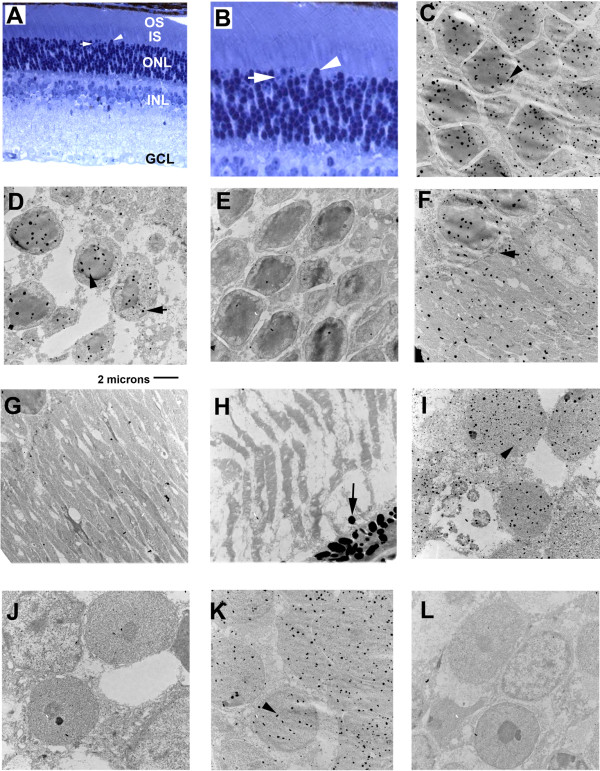
**Nano-gold TEM visualization of FIZ1's subcellular distribution in the P-35 mouse neural retina**. **A**) Light micrograph (400X) of the adult mouse neural retina, illustrating the relative layers and their location: the Ganglion Cell Layer (GCL), whose neurons form the optic nerve; the Inner Nuclear Layer (INL), nuclei of bipolar, horizontal and amacrine cells; the Outer Nuclear Layer (ONL), containing photoreceptor nuclei; the photoreceptor Inner Segments (IS); and the photoreceptor Outer Segments (OS), the location of disk membranes and phototransduction proteins, including Rhodopsin. (Lowicryl embedded section, Toluidine blue stain.) **A) **and **B) **Rod nuclei display dense chromatin staining throughout most of their nuclear volume (arrowhead). Cones have dense chromatin staining in a much smaller proportion of their nuclear volume (arrow). **C) **Nano-gold labeling of FIZ1 in the central ONL (arrowhead). Rod photoreceptors dominate. **D) **FIZ1 labeling in rod (arrowhead) and cone (arrow) nuclei near the ONL/IS boundary. **E) **ONL, pre-adsorbed antigen control. **F**) FIZ1 labeling of the photoreceptor IS region. The outer limiting membrane at the ONL/IS interface is indicated (arrow). **G) **IS, pre-adsorbed antigen control. **H) **The OS region did not display label for FIZ1. Pigment granules from the Retinal Pigment Epithelium (RPE) are present (arrow). **I) **FIZ1 labeling of the GCL, nuclei are positive for FIZ1 (arrowhead). **J) **GCL, pre-adsorbed antigen control. **K) **The INL layer, nuclei are positive for FIZ1 (arrowhead). **L) **INL, pre-adsorbed antigen control.

Regions of the adult mouse neural retina are illustrated by the light micrographs in Figure 1A, B. The ganglion cell layer (GCL), Inner Nuclear Layer (INL), and the Outer Nuclear Layer (ONL) form the three major cell layers in the mature tissue. The photoreceptor Inner Segments (IS) and Outer Segments (OS, contain visual transduction proteins) are restricted to the outer-most layer of the neural retina. Note that rod photoreceptor nuclei in the ONL are extremely compact compared to neurons of the INL and GCL. Most of the photoreceptor nuclei represent rods, while about 3–5% are cones. Rod nuclear cross-sectional areas are almost entirely densely staining chromatin. Cone nuclei are localized on the outer-most edge of the ONL layer, viewed here just below the IS, and display a much smaller proportion of densely staining cross-sectional area in comparison to rod nuclei. Examples of a rod (arrowhead) and cone (arrow) nucleus are indicated in a higher magnification in Figure 1B.

Nano-gold labeling of FIZ1 protein was visualized by TEM in the adult (P-35) retina (Fig. 1C–L). FIZ1 protein was present in cell nuclei of the ONL (photoreceptor), INL and GCL layers. Both rod and cone photoreceptor nuclei were positive for FIZ1 labeling, which was throughout the nucleus (Fig. 1C, D). FIZ1 was also detected in the inner-segment region of photoreceptors (Fig. 1F), but was absent from the outer-segments (Fig. 1H). FIZ1 antibody also labeled cell nuclei in the GCL and INL (Fig. 1I, K). Negative controls, using the same FIZ1 antibody pre-blocked with a six-fold molar excess of antigen, displayed a loss of FIZ1 labeling (Fig. 1E, G, J, L).

**Figure 2:**
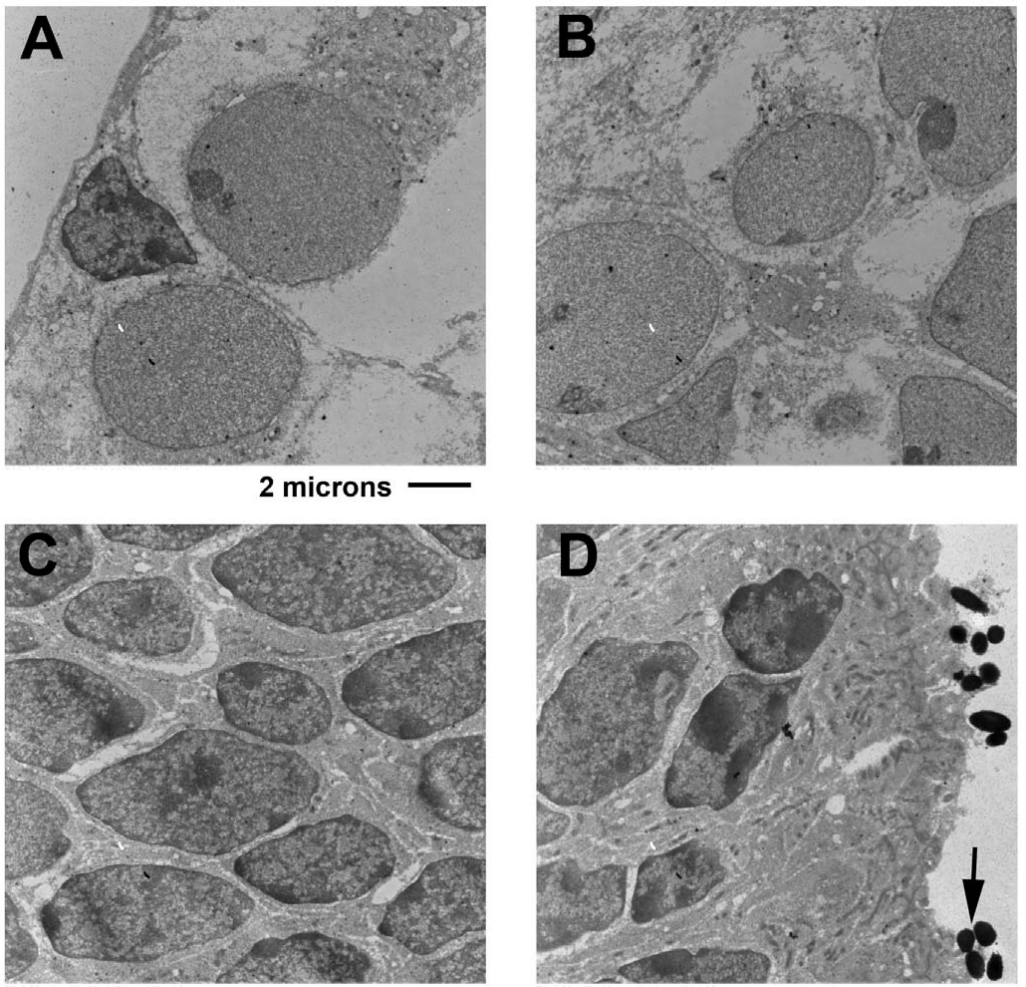
**Reduced FIZ1 concentration control: TEM-Immunogold of FIZ1 in the P-5 mouse neural retina**. As a control comparator for P-35 retina (See Figure 1), P-5 retinal sections were processed simultaneously with P-35 retina samples. The lower concentration of FIZ1 in P-5 retina was below detection sensitivity in the GCL **(A)**, the INL **(B)**, and the **C) **ONL (photoreceptor progenitor nuclei). **D) **Immature Inner Segments (IS) are in contact with pigment granules (arrow) from the RPE. Outer Segments (OS) are not present at P-5.

P-5 neural retina was processed simultaneously as a source of tissue that has at least 10-times lower endogenous FIZ1 protein content compared to adult neural retina (Fig. 2). Consistent with this, the lower level of FIZ1 at this stage of retinal development was not detectable with the nano-gold TEM method as used. This was the case for neurons in all layers present: the GCL, INL and ONL (Fig. 2A–D). Photoreceptor inner and outer segments were not present at the P-5 stage of ocular development.

### Co-IP of FIZ1 and CRX from retinal nuclear extracts

**Figure 3:**
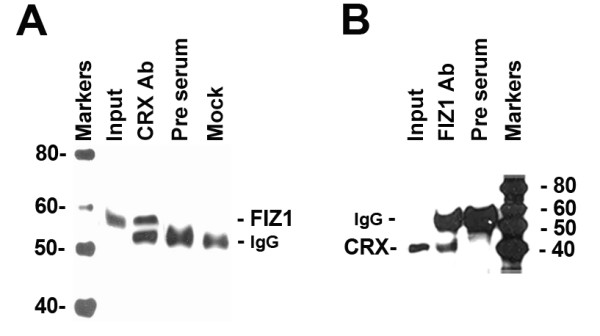
**Co-immunoprecipitation of native FIZ1 and CRX from bovine retina**. Immunoblots of Co-IP assays. **A**) Bovine retinal nuclear extract (300 μg protein) was incubated with anti-CRX antibody or pre-immune serum, and the immunoprecipitated proteins were analyzed with anti-bFIZ1 antibody. A mock sample (no extract) was included. **B**) The reverse Co-IP, using the anti-bFIZ1 antibody for precipitation and the anti-CRX antibody for detection.

To investigate if native FIZ1 complexes with CRX in neural retina, Co-IP experiments were carried out with bovine retina nuclear extract. Co-IP with the CRX antibody identified a band corresponding to FIZ1 (Fig. 3A). The full size gel format was used to separate FIZ1 from the heavy IgG band on immunoblots. Co-IP with pre-immune serum served as a negative control and identified the IgG heavy chain of the precipitating antibody. The reverse experiment was also performed, as Co-IP with the FIZ1 antibody, which identified a band corresponding to CRX on mini-gel immunoblots (Fig. 3B).

### Chromatin immunoprecipitation of FIZ1 at photoreceptor-specific gene promoters

**Figure 4:**
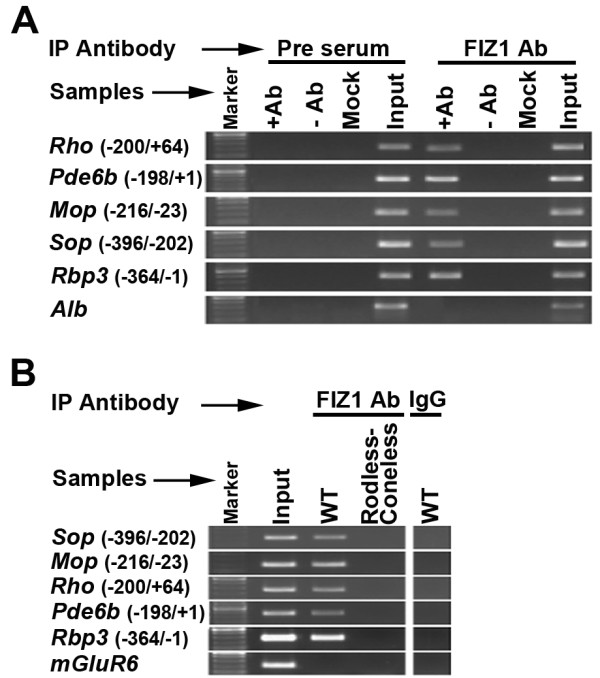
**FIZ1 is associated with photoreceptor gene promoters in photoreceptor neurons**. Combined agarose gel images. **A) **Chromatin immunoprecipitation (ChIP) assays were performed using the anti-bFIZ1 antibody. Normal IgG and no chromatin (mock) samples served as negative controls, whereas input DNA (without IP) served as positive controls. ChIP DNA was analyzed using PCR amplicons corresponding to the proximal promoter regions of the indicated photoreceptor-specific genes (*Sop*, *Mop*, *Rho*, *Pde6b *and *Rbp3*) and a non-photoreceptor gene (*Alb*, liver gene). **B) **ChIP assays were performed using the anti-FIZ1-1 antibody, and retinas of the following mouse strains: Wild-type (C57BL/6J), and Rodless/Coneless (*Rd*^*1*^). ChIP DNA was analyzed by PCR for promoter regions of key photoreceptor-specific genes (*Sop*, *Mop, Rho, Pde6b *and *Rbp3*) and non-photoreceptor genes (*mGluR6*, bipolar cells).

To determine if FIZ1 is present in the regulatory protein complex on the chromatin of photoreceptor genes in neural retina, we performed chromatin immunoprecipitation (ChIP) from wild type mouse retina with an antibody specific to FIZ1 (anti-bFIZ1). Results show that FIZ1 is associated with the proximal promoter regions of several genes that are known targets of CRX and NRL (Fig. 4A). These included the *Rho*, *Pde6b*, *M-Opsin*, *S-Opsin*, and *Rbp3 *(Inter Retinoid Binding Protein) gene promoters. Control ChIP assays, minus antibody or minus input DNA, were negative. Additional negative controls with the pre-immune serum IgG further demonstrated the specificity of the positive results obtained with the FIZ1-antibody. Assays did not detect any association of FIZ1 on the liver specific *Alb *(*Albumin*) gene promoter.

To determine if the association of FIZ1 with these gene promoters was specific to photoreceptor neurons, additional ChIP experiments were carried out using another antibody to FIZ1 (anti-FIZ1-1) that recognizes a conserved epitope sequence in domain-I of mouse, human and bovine FIZ1 (Fig. 4B). Neural retina tissue from two mouse strains, rodless/coneless and wild-type, were compared. Rodless/coneless mouse retinas still possessed non-photoreceptor neurons of the inner nuclear layer (bipolar, amacrine and horizontal cells) and the ganglion cell layer (ganglion and displaced amacrine cells); only the outer nuclear layer (photoreceptors) was absent. FIZ1 occupied the promoter regions of photoreceptor-specific genes in wild-type mice, but not in rodless/coneless mice (Fig. 4B). FIZ1 did not occupy the promoter region of *mGluR6*, which is expressed in bipolar cells, in either retina type. These results suggest that FIZ1 association with photoreceptor-specific gene promoters is specific to photoreceptor neurons.

### FIZ1 presence in NRL/CRX protein complexes bound to their responsive DNA motifs

**Figure 5:**
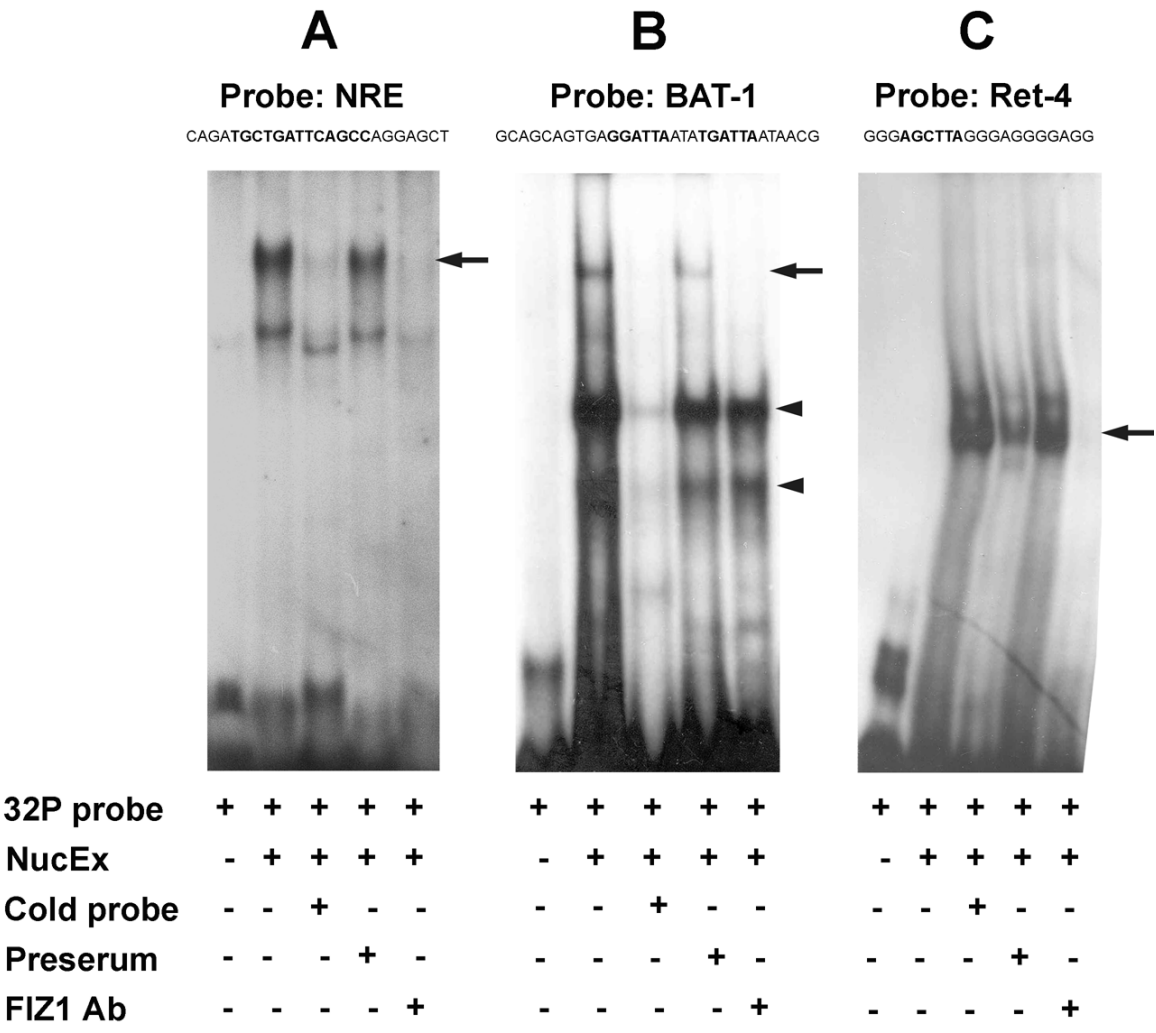
**FIZ1 is part of retinal protein complexes that bind to NRE, BAT-1 and Ret-4 elements from the *Rho *proximal promoter**. The [^32^P]-labeled oligonucleotide probes NRE, BAT-1, and Ret-4 were incubated with bovine retinal nuclear protein extract (NucEx), followed by nondenaturing PAGE. A) NRE: The binding site for the NRL bZIP domain is shown in bold face type. Removal of a shifted complex by antibody to FIZ1 is indicated (arrow). B) BAT-1: The binding sites for the Homeo-Domain of CRX are shown in bold face type. Removal of a shifted complex by antibody to FIZ1 is indicated (arrow). C) Ret-4: The core binding site for the CRX-HD is shown in bold face type. Removal of a shifted complex by antibody to FIZ1 is indicated (arrow). Presence of components are as indicated (+/-). Competition for each probe with unlabeled oligonucleotide validated the specificity of the band shift. Anti-FIZ1, or preserum IgG, was added after complex formation.

To determine if FIZ1 is part of retinal nuclear protein complexes that bind to DNA elements recognized by NRL and CRX, we performed electrophoretic mobility shift assays (EMSA) using bovine retina nuclear protein extract and ^32^P-labeled probes derived from conserved NRL- or CRX-responsive elements from the mammalian *Rho *proximal promoter region [5,21]. Previous EMSA results showed that bovine nuclear extracts produced two major shifted bands on a probe containing the NRL-Response Element (NRE) [21]. The NRE probe sequence and the NRL binding site are shown in Figure 5-A. In our assay, this probe was shifted in a characteristic pattern as previously published[21]. The higher shifted DNA-protein complex known to contain NRL[21] was efficiently removed by incubation with affinity-purified antibody (rabbit IgG) specific for FIZ1 (anti-bFIZ1), after complexes were allowed to form (indicated by the arrow). Control IgG (preimmune, rabbit IgG) did not remove any of the native protein-DNA complexes. As previously seen by Rehemtulla et al., 1996, competition with cold probe was more effective on diminishing the higher shifted complex. These results suggest that FIZ1 is a part of the NRL protein complex bound to NRE.

Likewise, EMSA was performed with two other probes, BAT-1 and Ret-4 [5], derived from the *Rho *promoter containing core sequences bound by the CRX Homeodomain (HD) (Figure 5-B and 5-C). BAT-1 has two elements that closely match the consensus binding sites for the K50-type HD (TAATCC and TAAGCT) [29-31], while Ret-4 has only one low affinity site for CRX. Our EMSA assays with both of these probes produced characteristic patterns reported previously with recombinant CRX-HD [5].

Figure 5B shows EMSA results with the BAT-1 probe and bovine retina nuclear protein extract. Two major shifted bands were seen, correspond to the "dimer" and monomer" of CRX reported previously [5] (indicated by arrowheads). In addition, we also detected a third protein complex running at a higher position than the two major CRX bands. Addition of an FIZ1 antibody to the pre-bound protein-DNA complexes abolished this third shifted band (indicated by the arrow) without affecting the two lower bands. Control IgG (preimmune, rabbit IgG) did not have any effect on the shifted bands. In contract, EMSA with the Ret-4 probe showed a doublet band shifted with retinal nuclear protein (Fig. 5C), corresponding to a protein complex containing CRX bound to a low affinity recognition site. This band (arrow) was efficiently removed by treatment with the FIZ1 antibody, suggesting that FIZ1 is a part of the CRX protein complex. Control IgG (preimmune, rabbit IgG) did not remove this band shift.

### Direct interaction of FIZ1 with CRX

**Figure 6:**
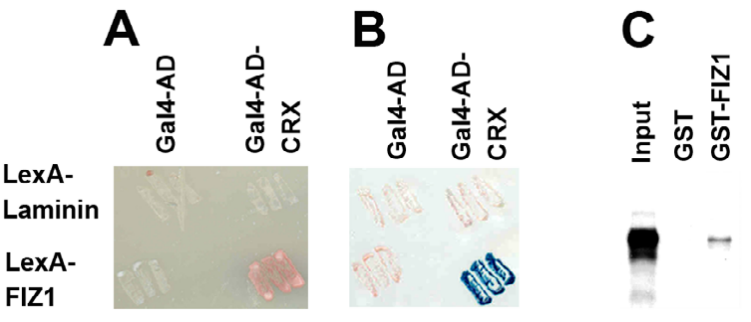
**FIZ1 can interact directly with CRX**. Yeast (L40) were transformed with plasmids that express LexA-hybrid proteins (pHybLex/Zeo-bFIZ or pHybLex/Zeo-Laminin) combined with Gal4-AD-hybrids (pACT2 or pACT2-bCRX). LexA-Laminin and Gal4-AD were negative controls. Protein interactions were tested by **A) **growth on minus-His medium, and **B) **β-galactosidase activity by filter lift assay. **C) **Autoradiogram of GST pull-down assays. ^35^S-CRX protein (input) was prepared by *in vitro *transcription/translation and processed with glutathione-Sepharose-GST-bFIZ1 or glutathione-Sepharose-GST, using equal amounts of GST-bFIZ1 or GST protein.

Since proteins known to complex with photoreceptor-specific promoters display multiple protein interactions, we wanted to determine if FIZ1 is capable of direct binding to CRX. We used two different methods: yeast two-hybrid and GST pull-down assays. Using the yeast two-hybrid method, L40 yeast cells were sequentially transformed with the desired bait- and prey-vector combinations. Potential protein interactions were evaluated with two interaction reporters: growth on minus-His medium (Fig. 6A) and expression of β-galactosidase (Fig. 6B). Double transformants with LexA-bFIZ1 + Gal4AD-bCRX were positive for interaction by growth on minus-His plates and by β-galactosidase filter lift assay. Control combinations for non-specific interactions were negative with both reporters (LexA-Laminin + Gal4AD-bCRX, and LexA-bFIZ1 + Gal4AD).

GST pull-down assays confirmed the direct interaction of FIZ1 and CRX (Fig. 6C). ^35^S-CRX was tested for binding to glutathione-Sepharose-GST-bFIZ1 compared to the glutathione-Sepharose-GST control. Assays contained equivalent amounts of GST and GST-bFIZ1. ^35^S-CRX bound to glutathione-Sepharose-GST-bFIZ1 when compared to glutathione-Sepharose-GST.

### FIZ1 interaction potentiates CRX-mediated promoter activation

**Figure 7:**
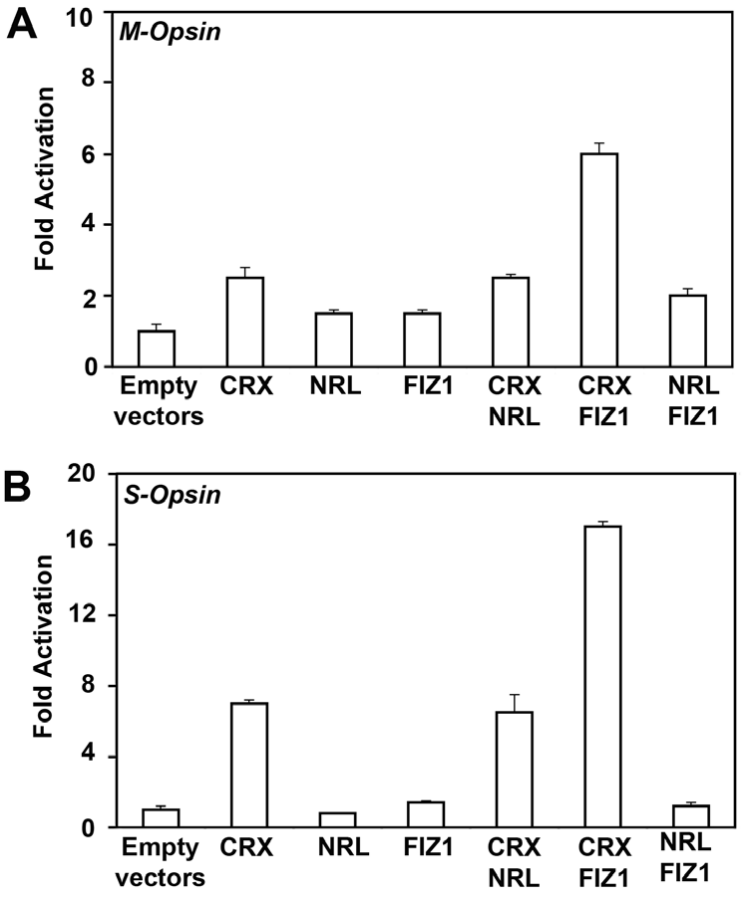
**FIZ1 alters the CRX-mediated activation of two Opsin promoters**. Two CRX-activated proximal promoters were used from the photoreceptor genes: *M-Opsin *and *S-Opsin*. Firefly luciferase activity was normalized to *Renilla*-luciferase activity (pRL-CMV). Fold Activation is relative to empty expression vectors. **A**) the *M-Opsin *promoter luciferase-reporter construct, *Mop250-Luc*, or **B**) the *S-Opsin *promoter luciferase-reporter construct, *Sop552-Luc*. Triplicate wells of CV1 cells were co-transfected with expression vectors for CRX, FIZ1, and NRL in the combinations indicated. CRX could activate both test promoters, relative to empty vectors. NRL or FIZ1 alone had no activation effect on either promoter. NRL combined with CRX did not alter the activation potential from that of CRX alone. FIZ1 combined with CRX significantly altered the CRX-mediated activation of both test promoters, when compared to CRX alone. Bars represent ± SD (n = 3). Statistical comparisons are described in results.

To determine if FIZ1 interaction with CRX could have any effect upon CRX-mediated activation of a test promoter, we employed a co-transfection luciferase-reporter assay system. Two test promoters were selected that represent photoreceptor-specific promoters that are capable of activation by CRX alone [9]. CV-1 cells were transfected with *M- *or *S-Opsin *reporter plasmids (firefly-luciferase) and expression plasmids for NRL, CRX and FIZ1. FIZ1 significantly altered the CRX-mediated activation of both test promoters. For the *M-Opsin *promoter, CRX alone caused a 2.5-fold activation relative to empty vectors (p < 0.01, Tukey Kramer post analysis) (Fig. 7A). Neither FIZ1 nor NRL alone had significant effect compared to empty vectors. FIZ1 combined with CRX increased the activation of the *M-Opsin *promoter, to 6-fold, relative to empty vectors (p < 0.01). The effect was to more than double CRX's activation potential.

CRX alone activated the *S-Opsin *promoter 7-fold (p < 0.01) (Fig. 7B). FIZ1 and NRL alone had no significant effect. As for the *M-Opsin *test promoter, FIZ1 combined with CRX more than doubled CRX's activation of the test promoter (p < 0.01).

### Association of FIZ1 with the Rhodopsin gene correlates to the increased presence of transcriptionally active RNA Polymerase-II *in vivo*

ChIP results indicated that FIZ1 is present at several photoreceptor-specific promoters *in vivo*; therefore, we next asked if the quantity of FIZ1 present at the *Rho *promoter is increased in adult neural retina (P25) compared to P3 neural retina? *Rho *is a fundamental marker of photoreceptor maturation; therefore, Q-ChIP analysis was performed to compare the binding of both FIZ1 and actively transcribing RNA-polymerase-II (Pol-II-S2) at the *Rho *gene. FIZ1 ChIP amplicons targeted the *Rho *proximal promoter region, while Pol-II-S2 ChIP amplicons targeted a site within Intron-2 of the gene.

**Figure 8:**
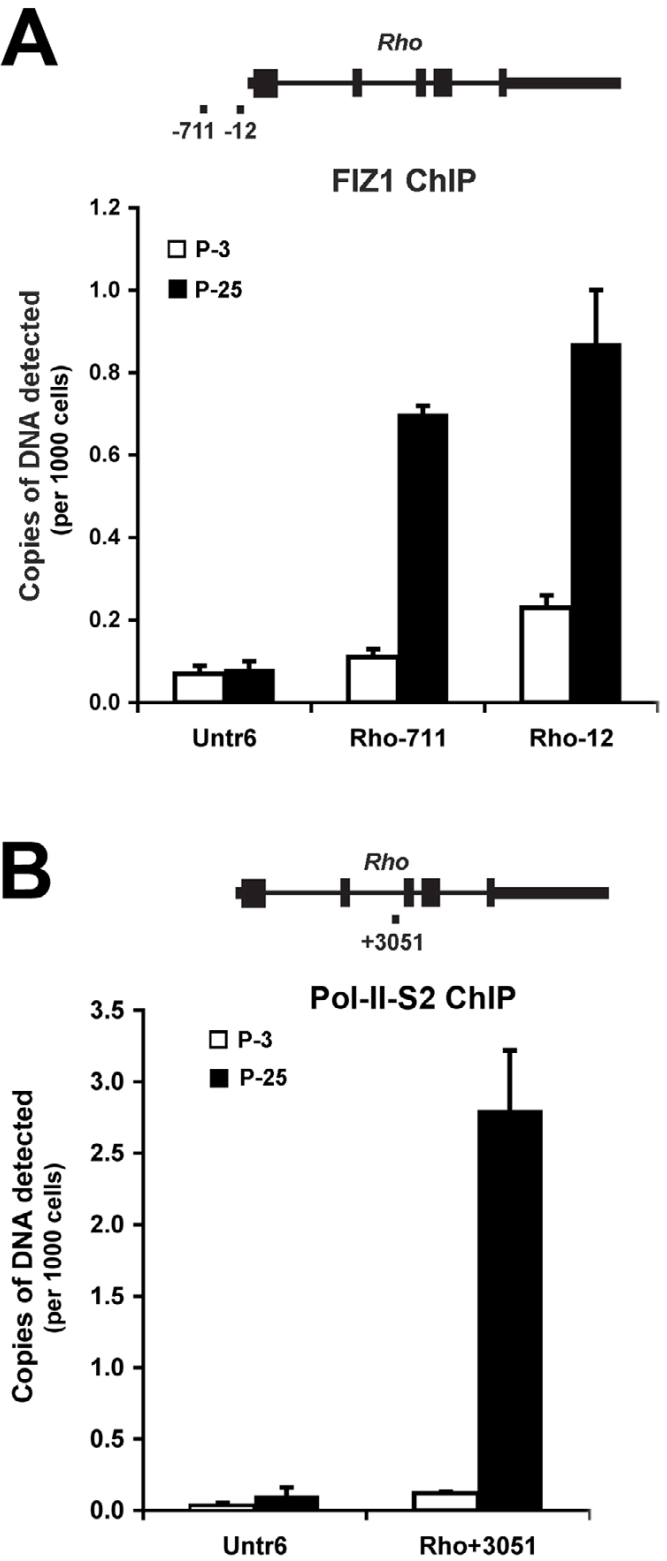
**Comparison of FIZ1 recruitment and activated Pol-II association, at the *Rhodopsin *gene in P-3 and P-25 retina**. ChIP was performed using antibodies specific for FIZ1 and the actively transcribing form of RNA Polymerase-II (Pol-II-S2). Protein binding is proportional to the amount of target DNA captured by ChIP, and is shown as copies of target DNA detected per 1000 cell equivalents of input DNA. Results were normalized for chromatin input and PCR efficiency. Error bars indicate standard deviation (n = 3). **A) **FIZ1: Q-PCR of two targets in the *Rho *proximal promoter (-711, -12), as illustrated in the diagram of the *Rho *gene. An untranslated region (*Untr6*), on chromosome-6, provided a reference for binding (negative control), and was not different comparing P-3 to P-25 neural retina. FIZ1 recruitment to the *Rho *promoter was significantly increased in P-25 neural retina compared to P-3 retina. **B) **Pol-II-S2: Q-PCR of a target inside intron-2 of the *Rho *gene (+3051), and the untranslated region (*Untr6*). Results confirm an increased amount of actively transcribing Pol-II in the transcriptional region of *Rho *in P-25 retina compared to P-3 retina.

The relative quantity of FIZ1, associated with the regulatory protein complex on the *Rho *promoter, was significantly elevated (7-fold) in P-25 neural retina compared to P-3 neural retina (Fig. 8A). FIZ1 association with this regulatory complex was significantly higher than for the untranslated control region (Untr6) in both P-3 and P-25 neural retina. In this untranslated control region, there was no difference in the relative association of FIZ1 comparing P-3 to P-25 neural retina.

The relative binding of transcriptionally active RNA polymerase-II (Pol-II-S2), monitored at a position about 3 Kb downstream from the *Rho *transcriptional start site, was significantly higher in the mature neural retina. Pol-II-S2 association was 23-fold higher on chromatin from P-25 neural retina compared to P-3 neural retina (Fig. 8B). There was no significant difference in the association of Pol-II-S2 in the untranslated control region (Untr6) comparing P-3 to P-25 neural retina.

## Discussion

Several lines of initial evidence have suggested that the FIZ1 protein can exist in both the cell's nuclear and cytoplasmic compartments in cultured cells and in neural retina [11-13]. We desired to visualize FIZ1 location in the neural retina at the subcellular level. Current improvements to TEM-immunogold techniques, particularly the use of smaller nano-gold antibody conjugates and silver-enhancement, provide clear visualization of nano-gold particles and lower background in electron micrographs of mammalian retina [14]. Ultra-thin sections also expose the dense chromatin matrix of the very compact nuclei of photoreceptor cells in the outer-nuclear layer.

Nano-gold TEM micrographs show that FIZ1 protein is present in the nuclei of all mature neurons in the ganglion, inner-nuclear and outer-nuclear layers (i.e. photoreceptors). This included nuclei of both rod and cone photoreceptors. Labeling of FIZ1 was present throughout the entire area of photoreceptor nuclei. While previous immunofluorescence study showed a distribution of NRL throughout rod-photoreceptor nuclei in human retina [32], the photoreceptors of nocturnal species (mouse) have compact nuclei with an inverted pattern of chromatin density. In mouse, dense chromatin fills the central nucleus, leaving lower density chromatin at the periphery. Evidence is mounting that this periphery corresponds to euchromatin, where active photoreceptor-specific genes are located. Fluorescent labeling of the mouse *Rho *gene has demonstrated a location in the periphery of most photoreceptor nuclei [33].

Some immunohistochemistry reports show detection of CRX throughout photoreceptor nuclei (mouse) [34,35], while immunofluorescence labeling with confocal imaging shows an intense peripheral labeling for CRX [9,33]. Immunofluorescence of NR2E3 in the mouse retina also shows an intense labeling in the nuclear periphery [9,10,36]. Mice expressing a fusion protein of GFP with OTX2, another transriptional activator present in photoreceptors, display a ring-like distribution of the GFP tag in their photoreceptors [37]. It is clear that this region correlates with the expression of active genes in mouse photoreceptors.

Recent reports also indicate that NRL and CRX have roles in the transcriptional repression of cone-specific genes in addition to their roles as transcriptional activators of rod-specific genes. The ectopic expression of NRL in mouse cone progenitors, results in the conversion of cone-precursors to rod photoreceptors [38]. CRX is also present on cone-opsin promoters in rod photoreceptor cells, and NR2E3 inhibits the CRX-mediated activation of cone-opsin promoters, *in vitro*[9,10]. The later could explain why NR2E3 deficient mice develop extra cone-opsin expressing photoreceptors in the form of hybrid cone-rod photoreceptors [39]. As such, NRL and CRX, two transcription factors that can bind FIZ1, could recruit FIZ1 to both euchromatin and heterochromatin regions of mouse photoreceptors.

Significant labeling of FIZ1 was also present in the inner segments of photoreceptors in mature retina. The packing of photoreceptor cells to high density is facilitated by the location of ribosomes and mitochondria in the inner-segments. As the location for much of the protein synthesis in photoreceptors, many proteins should be present in this region. We did not detect FIZ1 by immunogold staining in ultra thin sections of P-5 retina, which is likely due to the low sensitivity of this particular technique and a much lower level of FIZ1 protein in P-5 retina. This is consistent with previous findings, showing that FIZ1 concentration in immature neural retina is less than 10% of that found in the mature tissue [13]. The inability to detect this lower level of FIZ1 does not indicate that FIZ1 is absent. The more sensitive Q-Chip analysis confirmed this. FIZ1 association with the *Rho *promoter at P-3 was much lower than at P-25; however, the level of FIZ1 at P-3 was detectable above the background, as represented by an untranslated control region on the same chromosome (Chr-6).

Previously we have shown that FIZ1 could alter activation of a *Rho *test-promoter, suggesting that FIZ1 could have some direct or indirect interactions with CRX in addition to NRL [13]. CRX is present in all rod and cone photoreceptors, placing FIZ1 in a biologically relevant location for interaction with CRX. Our co-IP experiments from bovine retina nuclear extract, confirmed that native FIZ1 and CRX could be isolated as part of the same nuclear protein complex. Co-IP with antibody to FIZ1 captured CRX from nuclear protein preparations. Conversely, antibody to CRX co-precipitated FIZ1.

Peng and Chen (2005) have reported that NRL, CRX and NR2E3 are part of the transcriptional complex on several photoreceptor-specific genes *in vivo*, including *Rho*, *Pde6b*, *Rbp3*, *M-Opsin *and *S-Opsin *[17]. Our chromatin immunoprecipitation results indicated that FIZ1 is also associated with the proximal promoter regions of these genes. Our results here, and those previously reported, indicate that FIZ1 can alter the activation potential of NRL and/or CRX at four of these promoters *in vitro*: *Rho, PDE6B, M-Opsin*, and *S-Opsin*. Additionally, ChIP analysis found that FIZ1 occupies the regulatory complex of photoreceptor-specific genes in wild-type retina, but not in rodless/coneless mice (rd^1^). This result indicates that FIZ1 recruitment to the regulatory protein complex at photoreceptor-specific genes is an event specific to adult photoreceptors. While FIZ1 is present in bipolar and ganglion cells, it is not associated with photoreceptor-specific gene promoters in these neurons.

Our EMSA results indicate that FIZ1 is also a component of retinal nuclear protein complexes that can bind to the NRL-binding element (NRE), and the two CRX-binding elements (BAT-1, Ret-4). NRL binds to an extended AP-1 like element, the NRE, in the *Rho *gene's proximal promoter region [21,40,41]. The CRX homeodomain can bind to two promoter elements, BAT-1 and Ret-4, that flank the NRE [42]. Historically, the Ret-4 element was used in a yeast 1-hybrid survey to discover CRX from a bovine retina library [42].

Rehemtulla et al., demonstrated that the NRE probe is shifted into two major complexes with bovine nuclear protein extract, and the higher shifted complex can be removed by antibody to NRL [21]. In our tests here, FIZ1 antibody was also able to remove the larger probe/protein complex. While this element is relatively short and will be covered by NRL, stable protein-protein interactions could involve non-DNA binding proteins in the overall complex. FIZ1 is capable of direct and stable interaction with NRL *in vitro*; therefore, FIZ1 may associate with the NRE element indirectly through its interaction with NRL. This would be consistent with the fact that the higher shifted band, diminished by incubation with antibody to FIZ1, is also disrupted by antibody to NRL [21].

Similarly, it is conceivable for FIZ1 to associate indirectly with CRX-binding sites (BAT-1 and Ret-4) by association with CRX. BAT-1 has two core elements that can bind the K50-type HD of CRX [29,30]. Consistent with the presence of two potential binding sites, Chen et al., have demonstrated that the BAT-1 probe can be shifted into two major bands by recombinant CRX-HD [5]. The BAT-1 site is crucial for CRX mediated activation of the *Rhodopsin *proximal promoter [43].

Here, using bovine retina nuclear extract, two major shifted bands were also seen, similar to the pattern reported for recombinant CRX-HD. It is possible that these major shifted bands represent the association of one and two molecules of CRX. In our case, a third higher shifting faint band was removed after complex formation by treatment with FIZ1 antibody, while the two major bands remained. This suggests FIZ1 was not required for formation of the smaller complexes.

In contrast to BAT-1, the Ret-4 probe contains one K50-type HD binding element, and shifts to a doublet band with bovine retina nuclear extract [5,42]. Our EMSA here, with bovine retina nuclear extract, also resulted in a single major shifted complex. This intense band was efficiently removed by FIZ1 antibody, suggesting the presence of FIZ1. The disruption of only a higher shifting faint band with the BAT-1 probe, suggests that there could also be a different affinity for FIZ1 between a CRX dimer and monomer. This is interesting in the context of the *Rho *promoter, where the potential CRX-dimer binding site (BAT-1) and the monomer-binding site (Ret-4) flank the NRE site.

What could be the benefit of two different CRX-binding sites in such a small region? There is evidence that several other proteins interact with CRX at the *Rho *promoter, including NRL, NR2E3 and BAF [8,9,34]. Two CRX binding elements, binding in different stoichiometry, may provide a mechanism to have different CRX molecules participating in different protein interactions.

Our Co-IP and EMSA results indicated that FIZ1 might interact directly with CRX; therefore we explored this with yeast two-hybrid and GST pull-down assays. Both assay methods confirmed the ability for a direct and stable interaction of FIZ1 and CRX *in vitro*. This suggests that FIZ1 has at least two interaction partners in photoreceptor neurons (NRL and CRX) that could recruit it into transcription factor complexes on photoreceptor specific genes. Multiple protein-protein interactions are consistent with our knowledge of other proteins that complex at the *Rho *promoter. CRX participates in interactions with NRL and NR2E3, resulting in activation of rod genes and repression of cone genes [8,9]. NR2E3 also has multiple interactions, including CRX and the circadian clock protein NR1D1 [20].

While we previously demonstrated that FIZ1 could modify NRL's transactivation potential at a *PDE6B *test promoter, it only modified transactivation potential at the *Rho *promoter when both NRL and CRX were present [13]. To examine FIZ1 effect on CRX, we used two test promoters that are activated by CRX, but not NRL: *M- *and *S-Opsin *[42]. Alone, FIZ1 effect on the activation of either promoter was insignificant compared to empty vectors. However, FIZ1 was able to modify CRX's transactivation potential at both test promoters by over 100% of the activity seen with CRX alone. It is possible that FIZ1's presence could influence the overall activation potential of promoter complexes involving CRX.

The scale of FIZ1's effect on CRX-mediated activation is biologically significant. Mature photoreceptors must continue to precisely regulate the average expression of genes like *Rho *to maintain viable photoreceptors. In mouse neural retina, Rhodopsin mRNA levels vary at least 25% in a circadian fashion, to coordinate with the rhythm of outer-segment formation [44]. Heterozygous knockout of *Rho *reduces its average expression by 50%, which results in a slow degeneration of photoreceptors. Likewise, transgenic mice with elevated expression of normal Rhodopsin, also have a slow photoreceptor degeneration [45].

Developmentally, the increased availability of FIZ1 does concur with maturation of the neural retina and the activation of many photoreceptor-specific genes. While mRNAs for *M- *and *S-Opsin *are detected a few days earlier than for *Rho *[7], the initial formation of photoreceptor outer-segments of both rods and cones begins in the maturation period. This increases the demand for all three Opsin proteins as well as Pde6b, Arrestin, and other phototransduction proteins.

Test promoters are not perfect models for native gene promoters in their chromatin context, and FIZ1 can promote or inhibit NRL/CRX mediated activation of the *Rho *promoter *in vitro*, depending on the relative amount of FIZ1 compared to NRL and CRX [11,13]. With this in mind, it would be premature to state unequivocally whether FIZ1's role is that of promoting activation of these promoters *in vivo*, or if it may have a role in maintaining a precise level of their expression in the mature retina.

Our ChIP analysis indicates that FIZ1 is part of the regulatory protein complex on genes that become more active in adult photoreceptors. *Rho *is a fundamental marker of photoreceptor-specific gene expression; its expression increases dramatically as photoreceptor neurons mature to the adult state [13]. Is there an increase in the relative quantity of FIZ1 recruited to the *Rho *promoter complex in the adult retina? This would be expected if a prior association of NRL and CRX recruits FIZ1. Relative association of FIZ1 at the *Rho *promoter region was 7-fold greater in adult neural retina (P-25), as compared to the immature tissue (P-3) when only a small fraction of photoreceptor precursors are starting to express *Rho*.

The increased association of FIZ1 at the *Rho *promoter also correlated with a 23-fold increase in the amount of actively transcribing Pol-II in adult neural retina (P-25) compared to the immature tissue (P-3). The total Pol-II associated with the *Rho *proximal-promoter region increases during maturation of the mouse retina [7]. While the presence of Pol-II in the promoter region often correlates to transcription, it is possible for a gene to be pre-loaded with inactive Pol-II, as recently reported for many genes in human embryonic stem cells [46]. Our assays here specifically monitored actively transcribing Pol-II (serine-2 phosphorylated) in intron-2, about 3 Kb downstream of the *Rhodopsin *gene's transcription start site.

The increased expression of several photoreceptor genes, during maturation of photoreceptors, certainly involves regulation at the level of transcription. Rhodopsin and Arrestin mRNA levels increase dramatically during this process, and this is documented in mammalian species that complete retinal development postnatal (mouse) or prenatal (bovine) [44,47]. A previous study of bovine retinal development, using nuclear run-on assays, suggested that elevated transcription has a prominent role during this maturation period [47]. Our quantitative measurement of transcriptionally active Pol-II within the *Rho's *transcript region, confirms that the amount of activated Pol-II enzyme is elevated.

## Conclusion

Recently, Peng and Chen (2007) demonstrated that CRX and NRL are not present on the *Rhodopsin *promoter until the start of *Rho *expression, even though both transcription factors are abundant in rod-precursors before this time [7]. This suggests that additional factors are involved with the formation of a stable regulatory protein complex on these promoters as the retina matures to the adult state. Our results suggest that FIZ1 is one of the additional proteins recruited by NRL and CRX to these transcription factor complexes, and both its bioavailability and association at the *Rho *promoter are increased substantially as the neural retina matures. While FIZ1 does not appear to be a direct transcriptional activator, it can significantly alter the CRX-mediated activation of cone Opsin promoters *in vitro*.

In a mouse hematopoietic cell line, engineered to over express FLT-3 (FMN like tyrosine kinase) and FIZ1, FIZ1 association with FLT-3 was reported to increase upon receptor activation. While the downstream consequences are not known, those studies also found FIZ1 to be present in both the cytoplasm and nucleus [12]. Our results here showing FIZ1 recruitment to gene promoter complexes, suggest a potential role in signal transduction between receptors and genes. While the expression of FIZ1 is ubiquitous in mammals, the reported interactions thus far are tissue specific. Additional tissue-specific interactions await discovery for organs not yet explored regarding FIZ1 function. The data gathered so far justifies the production of animal models for over and under expression of FIZ1, which will be required to explore its tissue-specific functions.

Q-ChIP was successfully applied to neural retina tissue to directly detect an increase in the transcriptionally active form of RNA-Polymerase-II moving through *Rho's *transcriptional region. This analysis provides a novel way to directly quantify the activation state of a gene in the context of neural development, and is applicable to any gene of interest. Finally, we note that the presence of FIZ1 in the nuclei of all mature retinal neurons suggests that FIZ1 may also be recruited to non-photoreceptor specific genes that become active in the adult retina. Further studies to find more target genes for the FIZ1 protein will shed light on this possibility.

## Abbreviations

FIZ1: (Flt-3 Interacting Zinc-finger); NRL: (Neural Retina Leucine-zipper); CRX: (Cone-Rod homeoboX); *Rho*: (Rhodopsin); *Pde6b*: (Phosphodiesterase 6 beta subunit); NR2E3: (PNR/photoreceptor-specific nuclear receptor); *Rpb3*: (Inter-Retinoid Binding Protein); *GluR6*: (metabotropic glutamate receptor subtype-6); ChIP: (Chromatin Immunoprecipitation); FLT-3: (FMS like tyrosine kinase -3).

## Authors' contributions

RM carried out Co-IP, GST pull down, yeast two-hybrid, and EMSA experiments, participated in design and execution of transfection promoter assays, and drafted the manuscript with KM. GP and SC designed and carried out PCR-ChIP and participated in manuscript editing. ZX carried out transfections and promoter assays and participated in analysis and editing of the manuscript. LD assisted with design of TEM immunogold methods, carried out TEM-Immunogold, and edited content of the manuscript. KM: developed the study, coordinated the project, assisted in design and execution of protein interaction experiments, promoter activation tests, and TEM-immunogold. KM also designed and managed the Q-ChIP tissue analysis and drafted the manuscript with RM. All authors read and approved the final manuscript.
